# Backward Phase Matching for Second Harmonic Generation in Negative‐Index Conformal Surface Plasmonic Metamaterials

**DOI:** 10.1002/advs.201800661

**Published:** 2018-08-31

**Authors:** Liangliang Liu, Lin Wu, Jingjing Zhang, Zhuo Li, Baile Zhang, Yu Luo

**Affiliations:** ^1^ School of Electrical and Electronic Engineering Nanyang Technological University Nanyang Avenue 639798 Singapore; ^2^ The Research Center of Applied Electromagnetics School of Electronic and Information Engineering Nanjing University of Information Science and Technology Nanjing 210044 China; ^3^ The Key Laboratory of Radar Imaging and Microwave Photonics Ministry of Education College of Electronic and Information Engineering Nanjing University of Aeronautics and Astronautics Nanjing 211106 China; ^4^ Division of Physics and Applied Physics School of Physical and Mathematical Sciences Nanyang Technological University Nanyang Avenue 637371 Singapore

**Keywords:** backward phase matching, efficient frequency mixing, high efficiency and ultrathin flexible geometry, negative‐index spoof plasmons, nonlinear metamaterials

## Abstract

Backward phase matching, which describes counterpropagating fundamental and harmonic waves in a negative‐index medium, is one of the most intriguing phenomena in nonlinear metamaterials. Predicted theoretically decades ago, however, it is still a challenging task to be applied for efficient second harmonic (SH) generation in a nonlinear metamaterial with ultrathin geometry and ultralow loss. Here, a negative‐index spoof plasmonic metamaterial is reported, which is composed of an ultrathin symmetrical corrugated metallic strips loaded with nonlinear active devices. The simulated and measured power spectra and surface near‐field distributions show that a peak SH signal can be generated at the backward phase‐matched frequency point in a 120° curved surface with high efficiency, thanks to the ultrathin flexible geometry, significant confinement effect, and large propagation length of the spoof surface plasmons. The results open new technological challenges from nano‐ and micro‐nonlinear photonics to science and engineering of compact, broadband, and efficient frequency‐mixing metamaterials and electromagnetic devices.

## 1 Introduction

Nonlinear optics is of tremendous importance to modern, light‐based technology.^1^ The recent uses of nonlinear metamaterials to enhance and control nonlinear response, such as frequency generation,^2^, ^3^, ^4^ parametric amplification,^5^, ^6^ bistability,^7^, ^8^ second harmonic generation (SHG),^2^, ^9^, ^10^ three‐ and four‐wave mixing,^11^ have promoted various applications ranging from laser‐based techniques^12^ and microscopy^13^ to optical and quantum information processing.^14^ However, the setups in those nonlinear processes mostly limited to subwavelength interaction lengths to avoid the destructive effects of phase mismatch. This greatly reduces the conversion efficiencies, thereby limiting the practical applications.

Backward phase matching, one of the most intriguing phenomena in nonlinear metamaterials, has attracted surging interests. Past studies are mostly focused on theoretical demonstrations,^9^, ^15^, ^16^ while in recent years, more attentions have been paid to the experimental verification of this phenomenon. However, the reported designs heavily rely on the double‐negative (the permittivity ε < 0 and the permeability *µ* < 0) magnetic metamaterial,^17^ which will induce significant loss and reflectance due to the magnetic response. To address this problem, we ask the question: Can we replace the lossy double‐negative materials with low‐loss single‐negative (ε < 0 and *µ* = *µ*_0_, *µ*_0_ is the permeability in free space) ones to realize highly efficient backward SHG? Lately, a new method based on a bulk metal–insulator–metal plasmonic waveguide, which is a single‐negative electric material, has been proposed to realize backward phase matching for SHG.^18^ Nevertheless, owing to the inherent large metallic loss and short propagation length associated with optical surface plasmons,^19^, ^20^, ^21^, ^22^, ^23^, ^24^ it is difficult to obtain high conversion efficiency of backward phase‐matched SHG within a short coherence length. Moreover, the inherent bulk 3D geometry limits their applications in the production of advanced nonlinear plasmonic functional circuits, devices, and systems integrated on flexible, stretchable, and biocompatible curved substrates. Therefore, the generation of efficient backward phase‐matched second harmonic (SH) in a nonlinear metamaterial with ultrathin geometry and ultralow loss is of great importance in practical applications. Our work aims at tackling the above challenges.

Highly conducting surfaces can support spoof surface plasmon modes when textured at the subwavelength scale.^25^, ^26^, ^27^ The unique ability of these spoof surface plasmons to emulate optical‐frequency surface plasmons at much lower frequencies has opened novel routes for wave control of terahertz^28^, ^29^, ^30^, ^31^ and microwave radiation.^32^ Recently, the versatility of these concepts has been substantially extended with the demonstration of a novel class of geometry‐induced modes supported by ultrathin and flexible metal films (the so‐called conformal surface plasmons (CSPs)).^33^, ^34^ which is proved to be a promising candidate for developing practical ultrathin surface circuitry and devices at microwave and terahertz frequencies due to its simplicity of the structures supporting CSPs and their inherent design advantages including deep‐subwavelength confinement and enhancement, low bending losses, broadband operation, and ultrathin flexible geometry.^35^, ^36^, ^37^, ^38^, ^39^, ^40^, ^41^, ^42^

**Figure 1:**
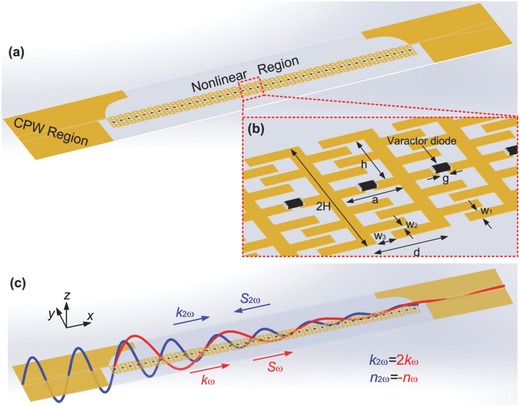
Schematic showing the backward phase‐matching effect in spoof plasmonic metamaterial. a) The proposed negative‐index nonlinear spoof plasmonic metamaterial. b) Enlarged view of nonlinear unit cells, where the period *d* = 3.5 mm, *a* = 2.8 mm, *h* = 3.0 mm, *H* = 4.4 mm, *w*_1_ = 0.6 mm, *w*_2_ = 0.2 mm, *w*_3_ = 0.8 mm, and *g* = 0.3 mm. c) Schematic diagram of the backward phase matching for SHG in the nonlinear spoof plasmonic metamaterial. The red‐ and blue‐colored lines represent the FF and SH waves, respectively.

In this work, we report a negative‐index nonlinear spoof plasmonic metamaterial to achieve the backward phase matching for efficient SHG. The proposed nonlinear spoof plasmonic metamaterial is composed of an ultrathin mirror‐symmetric corrugated metallic strip loaded with varactor diodes shown in **Figure** **1**. We have observed the dispersion relation of the antisymmetric mode with a negative slope and experimentally realized the backward phase‐matching condition at the frequencies of 3.62 and 7.24 GHz for the fundamental and SH waves with high conversion efficiency. We believe that these results can contribute to the development of more complicated nonlinear surface plasmonic circuitry for microwave and terahertz applications.

## 2 Negative‐Index Nonlinear Spoof Plasmonic Metamaterial

As a result of wave–matter interactions, the dispersion property includes most of the propagating information of the electromagnetic (EM) waves. When nonlinear wave mixing occurs in a waveguide instead of a bulk medium, the backward phase‐matching condition for SHG requires the wavevectors and the Poynting vectors of the fundamental frequency (FF) source (*k*_ω_, *S*_ω_) and the SH (*k*_2ω_, *S*_2ω_) to be identical and antiparallel to each other, respectively.^9^, ^16^ This means that in a nonlinear negative‐index metamaterial with refractive index *n*_ω_ > 0 and *n*_2ω_ < 0, both wavevector *k*_2ω_ = 2*k*_ω_ and refractive index *n*_2ω_ = −*n*_ω_ need to be satisfied simultaneously.

To achieve the backward phase matching in proposed spoof plasmonic metamaterial, two strategies should be addressed. First, according to the Maxwell's theory, deep‐subwavelength particles play a role of lumped elements in the microwave or optical circuits analogous to microelectronics.^43^ It is possible to construct a nonlinear material from the notion of circuit to unit cell by introducing tunable materials or tunable particles, which can be regarded as tunable lumped elements (details in Figure S1, Supporting Information). From this idea, considering the potential applications, each CSP unit is introduced into a variable capacity diode (model Skyworks SMV‐1231‐079LF^44^) which introduces nonlinear current–voltage dependence and results nonlinear electric dipole moment to each unit cell, and consequently a nonlinear spoof plasmonic metamaterial is realized.

**Figure 2:**
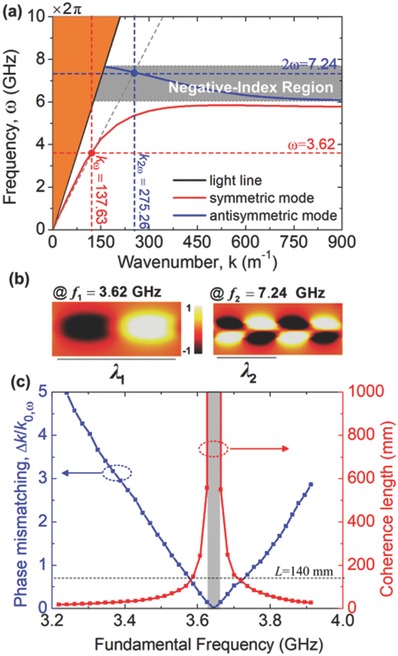
Operating point for backward phase matching in a spoof plasmonic waveguide. a) Dispersion relation of the nonlinear CSP to achieve backward phase‐matching condition, where the gray part represents the negative refractive index region. b) Electric‐field mapping for the symmetric and the antisymmetric modes respectively. c) Calculated degree of phase mismatch (Δ*k*/*k*_0,ω_, blue line) at different FFs and the corresponding coherence length (red line), *L* = 140 mm is the length of the nonlinear spoof plasmonic metamaterial we used (black dotted line).

Moreover, to achieve the negative‐index in the nonlinear spoof plasmonic metamaterial, a special CSP structure composed by a varactor‐loaded ultrathin mirror‐symmetric corrugated metallic strip with thickness 0.018 mm(≈λ/2300 when *f*_SH_ = 7.24 GHz) is designed, as shown in the nonlinear region in Figure 1a. Then, we numerically calculated the dispersion relations of the nonlinear CSP structure shown in **Figure** **2**a (details in Experimental Section). In which, the dispersion curves are decomposed into a lower‐ and a higher‐frequency band due to the close coupling effect, corresponding to the bonding and antibonding combination of the nonlinear CSP at both sides of the strip, respectively. The lower‐frequency branch (symmetric mode) has a positive slope, while the higher‐frequency branch (antisymmetric mode) shows a negative slope, leading to an antiparallel relation between the phase velocity *v*_p_ = ω/*k* and the group velocity *v*_g_ = dω/d*k*, and consequently, a negative‐index for the antisymmetric mode occurs. Thus, we set the refractive index for the FF wave (ω) to be positive and set the SH frequency (2ω) to be within the negative‐index band in this work.

When the backward phase matching occurs, the SH output grows toward the source of the fundamental wave, as shown in Figure 1c. It is worth noting that the dispersion relation of the nonlinear spoof plasmonic metamaterial is insensitive to the capacitance value of the varactor (details in Figure S2a,b, Supporting Information), which implies that the backward phase matching for SHG can be achieved by simply optimizing the geometry of the CSP structure and it is so important for the phase‐matching process in the negative‐index nonlinear spoof plasmonic metamaterial. By optimizing the geometry parameters of the nonlinear unit cell, we have located an operational point where the fundamental wave at ω_FF_/2π = 3.62 GHz with wavenumber *k*_FF_ = 137.63 m^−1^ in the symmetric mode is phase matched to the generated SH signal at ω_SH_/2π = 7.24 GHz (ω_FF_/2π and ω_SH_/2π are represented by ƒ_FF_ and ƒ_SH_ in the following work) with wavenumber *k*_SH_ = 275.26 m^−1^ in the antisymmetric mode, marked by the dotted lines in Figure 2a, respectively. To demonstrate the propagation characteristic of the fundamental and backward SH waves, the transverse electric‐field distributions at 2 mm above the surface of the plasmonic waveguide (zero‐voltage capacitance value *C*_0_ = 2.35 pF is used) at ƒ_FF_ = 3.62 GHz and ƒ_SH_ = 7.24 GHz have been obtained using the transient solver in the Microwave Studio of CST, as shown in Figure 2b. The two modes exhibit opposite field profiles, which lead to a vanishing mode‐overlapping factor for SHG. Figure 2c shows the coherence length *L*_coh_ = 2π/|Δ*k*| calculated according to the simulated dispersion curves in Figure 2a, where Δ*k* = *k*_2ω_ − 2*k*_ω_ and *k*_0,ω_ represents the wavenumber at the FF in free space. As expected, the coherence length for the backward SH is very large in the negative‐index band. The backward phase‐matching region, shown in gray in Figure 2c, represents the frequency range in which our nonlinear spoof plasmonic metamaterial acts as the nonlinear‐optical mirror.

## 3 Experimental Demonstration of Backward Phase Matching for SHG

**Figure 3:**
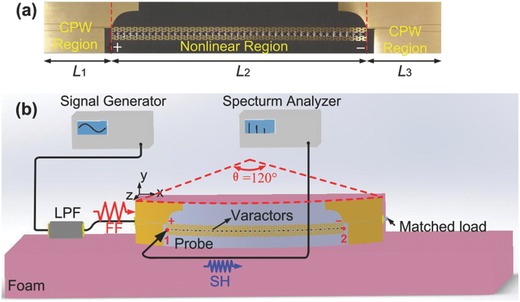
Experimental setup for detection of the backward phase‐matching effect. a) Photograph of the fabricated sample, where *L*_1_ = *L*_3_ = 40 mm, *L*_2_ = 140 mm. b) Schematic diagram of spectrum measurements for SHG in the negative‐index nonlinear spoof plasmonic metamaterial, where the fabricated sample was attached to a θ = 120° curved foam board.

To experimentally confirm the backward phase matching for SHG in the negative‐index nonlinear spoof plasmonic metamaterial, we fabricate a sample with 40 nonlinear unit cells shown in **Figure** **3**a, whose geometry parameters and the varactor loading scheme are the same as that listed in the simulation scenarios in Figures 1 and 2. To facilitate inject and measure the FF and SH waves, a coplanar waveguide of length *L*_1_ = 40 mm (*L*_3_ = *L*_1_ = 40 mm) with 50 Ω port impedance is employed. In the nonlinear region, the nonlinear CSP waveguide with length *L*_2_ = 40d = 140 mm is designed and connected to both sides of the coplanar waveguide. The whole length of the sample is 220 mm.

### 3.1 Spectrum Measurement

**Figure 4:**
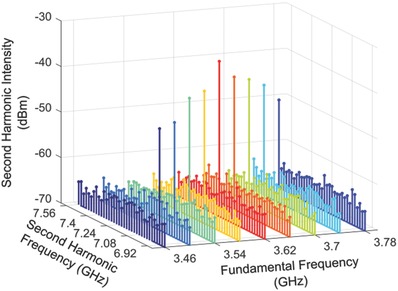
Frequency‐doubled signals emerging from the nonlinear spoof plasmonic metamaterial. Spectra from the nonlinear spoof plasmonic metamaterial at a series of *f*_FF_. The intensity of the pump light is maintained at a constant level. At each excitation frequency *f*_FF_, the output signal features a distinct peak at 2*f*_FF_.

As shown in Figure 3b, a schematic diagram of comprehensive setup is used for the power spectrum measurements of the SHG (details in Experimental Section). First, the nonlinear spectrum of the backward SHG signal at probe 1, which is positioned left of the nonlinear region and marked by the left red point in Figure 3b, is measured and shown in **Figure** **4**. The incident FF varies from 3.46 to 3.78 GHz with a step of 0.04 GHz, and the output power (intensity) of the microwave signal generator is maintained at a constant level of 10 dBm. We observe that each induced SHG spectrum features a distinct peak for each incident FF signal, and a maximum SHG intensity at the frequency of ƒ_SH_ = 7.24 GHz is identified, where the backward phase matching occurs.

**Figure 5:**
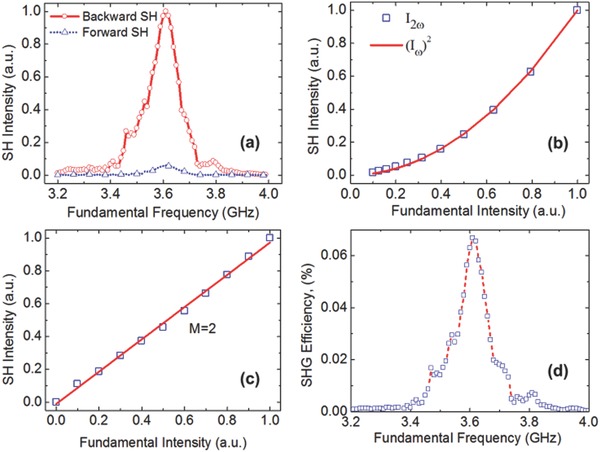
Backward phase‐matched SHG in the nonlinear spoof plasmonic metamaterial. a) Comparison of the backward and forward SHG intensities at a series of broadband incident FFs. b) Dependence of the induced SHG intensity on the externally applied intensity of the FF (ƒ_FF_ = 3.62 GHz) signal, which depicts an approximate quadratic relation of *I*_2ω_ ∝ (*I*_ω_)^2^. c) The logarithmic relation of the *I*_2ω_ and *I*_ω_ in (b). d) The conversion efficiency of the backward SHG at a series of broadband incident FF signals.

Then, to distinguish the forward and backward SHG, both the SHG powers in probe 1 and probe 2 (positioned on the right of the nonlinear region and marked by the red point in Figure 3b are measured when a broadband incident FF signals varied from 3.2 to 4 GHz with a step of 0.01 GHz, as shown in the red solid and blue dotted lines in **Figure** **5**a. Here, the chosen FF signals are intended to ensure the SHG within the negative‐index region of the nonlinear CSP waveguide in Figure 2a. Although a series of backward SHG signals are reflected and propagate toward to the opposite direction due to the impedance mismatch between the coplanar and the CSP waveguides. The SHG power collected in probe 2 is obviously significant weaker than that in probe 1 within the entire bands, indicating the effect from the forward SHG can be ignored. It can be obviously seen that a peak value of SHG power appears at *f*_SH_ = 7.24 GHz where the backward phase matching occurs. This means that a maximum backward SHG can be achieved at the phase‐matching frequency point in the nonlinear spoof plasmonic metamaterial. Moreover, we find that the measured SHG signals in Figure 5a are in a relatively broad frequency band. This is because when the length of the nonlinear spoof plasmonic metamaterial is long enough, i.e., Δ*k* · *L* ≥ 2π (where Δ*k* = |*k*_2ω_ − 2*k*_ω_| and *L* is the length of plasmonic waveguide), the SHs generated will add up destructively. In this case, we can only get efficient SHG over a very narrow frequency range where the phase‐matching condition is rigorously satisfied. However, in our case, Δ*k* · *L* < 2π over a relatively broad frequency band, from 3.58 to 3.71 GHz. Within this frequency range, the SHs generated will not completely cancel out each other, resulting in a relatively broadband backward SHG. As shown in Figure 2c, the coherent length is longer than the length of the nonlinear plasmonic waveguide from 3.58 to 3.71 GHz, indicating Δ*k* · *L* < 2π and therefore relatively strong SHG can be observed in this frequency range. We also want to highlight that this frequency band (i.e., 3.58 to 3.71 GHz) is consistent with the full width half maximum bandwidth shown in Figure 5a, demonstrating the validity of our experimental measurement. And thus, the backward phase‐matching effect occurring in the negative‐index nonlinear spoof plasmonic metamaterial therefore be demonstrated explicitly.

Like the conventional SHG, the SH intensity generated in a nonlinear waveguide of length *L* due to wave mixing from mode *i* at the fundamental wavelength λ_ω_ to mode *j* at the harmonic wavelength λ_2ω_ can be expressed as

(1) $$I_{2\omega }\propto \frac{8\pi^{2}L^{2}{\left(\chi^{\left(2\right)}\right)}^{2}}{\left|n_{\omega }^{2}n_{2\omega }\lambda_{\omega }^{2}c\varepsilon_{0}\right|}{\left[sin\left(\frac{\Delta kL}{2}\right)/\frac{\Delta kL}{2}\right]}^{2}I_{\omega }^{2},$$

where *L* is the length of nonlinear region, the superscripts ω and 2ω correspond to the fundamental and SH waves, respectively, *I* is the intensity, *n* is the refractive index, Δ*k* = |*k*_2ω_ − 2*k*_ω_| as defined previously, χ^(2)^ denotes the second‐order susceptibility and λ_ω_ is the wavelength of the incident fundamental wave, *c* and ε_0_ are the light velocity and permittivity of the free space, respectively. We measured the frequency‐doubled signals while varying the incident power of the FF wave. As shown in Figure 5b, the square points depict the measured backward SHG intensity, and the solid line is the fitting curve obtained by the least‐squares fitting method using the function *I*_2ω_ ∝ (*I*_ω_)*^M^*. As a result, the least‐squares fitting exponent is found to be *M* = 2, which agrees well with that in the expression (1). This result also further demonstrates that our measured results are accurate and reasonable. The curve in Figure 5c shows the same relation between the intensities of the FF and SH waves, in logarithmic unit using the relation function log(*I*_2ω_) ∝ 2log(*I*_ω_). This relation represents an approximate quadratic function, and the error is owing to that not all the backward SHG powers are collected by the probe and the effect of the back reflection of the forward SHG signals.

### 3.2 Near‐Field Measurement

To demonstrate the excellent field confinement and highly efficient propagation property of the backward SHG in the nonlinear spoof plasmonic metamaterial, the surface electric‐field distributions on the *xy* plane under different incident FF signals are measured using a near‐field scanning system (details in Experimental Section).

**Figure 6:**
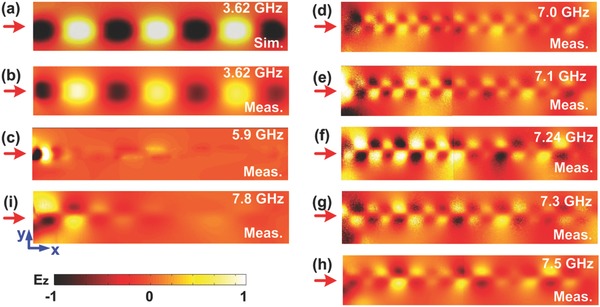
Observation of backward phase‐matching effect with electric‐field distributions. The a) simulated and b) measured |Ez| distributions on the *xy* plane at *f* = 3.62 GHz. c–i) The measured |Ez| distributions on the *xy* plane at *f* = 5.9, 7.0, 7.1, 7.24, 7.3, 7.5, and 7.8 GHz, respectively (the red arrows indicate the incident direction of the FF signals).

Using the experimental setup, the near‐electric‐field (|Ez|‐component) distributions obtained on the measurement plane with a step resolution of 0.5 mm both in the *x*‐ and *y*‐directions are displayed in **Figure** **6**. As shown in Figure 6a, the electric field at *f*_FF_ = 3.62 GHz is tightly confined around the corrugated metallic strips and shows agreement well with the simulated result in Figure 6b. This ensures the efficient FF signal propagation in the nonlinear spoof plasmonic metamaterial. We observe that the measured electric field at *f*_FF_ = 3.62 GHz is gradually weakened along *x*‐direction, verifying the propagation characteristic of the FF wave in the phase‐matching process in Figure 1c. We also measured some non‐phase‐matching frequency points such as *f*_SH_ = 7.0, 7.1, 7.24, 7.3, 7.5 GHz, respectively, aiming to demonstrate the backward phase‐matching effect in the nonlinear spoof plasmonic metamaterial from the perspective of the evolution of the electric‐field distributions. From Figure 6d–h, we notice that the |Ez| intensity is gradually increased from 7.0 to 7.24 GHz and decreased from 7.24 to 7.5 GHz. These results indicate that numerous scattering fields may emerge at non‐phase‐matching frequency points of 7.0, 7.1, 7.3, and 7.5 GHz, while being effectively suppressed at *f* = 7.24 GHz. Moreover, a maximum signal intensity generated when the phase matching occurs, which further verifies the backward phase matching for SHG in the nonlinear spoof plasmonic metamaterial. Furthermore, it should be noted that the CSP waves are not supported at *f* = 5.9 (see Figure 6c) and 7.8 GHz (see Figure 6i), respectively, which correspond to the bandgap bands of the nonlinear CSPs shown in Figure 2a.

## 4 Estimation of the Conversion Efficiency

We can estimate the conversion efficiency of the backward SHG process based on the above experimental condition in Figure 3b. In this work, we measured the powers of the incident FF and the induced backward SHG at probe 1 on the sample of nonlinear spoof plasmonic metamaterial and recorded them as *I*_ω_ (power in unit dBm, the same below) and *I*_2ω_, respectively. The overall conversion efficiency of the backward SHG can be estimated by

(2) $$\eta =\frac{I_{2\omega }}{I_{\omega }}\times 100\%.$$

Thus, the conversion efficiency of the backward SHG when the FF varies from 3.2 to 4 GHz with a step of 0.01 GHz is measured and calculated shown in Figure 5d. It shows that a peak value of η = 0.067% (*I*_ω_ = −6.94 dBm and *I*_2ω_ = −38.78 dBm) appears at 7.24 GHz where the backward phase matching occurs. This means that a maximum conversion efficiency of the backward SHG can be achieved at the phase‐matching frequency point in the nonlinear spoof plasmonic metamaterial, indicating a high‐efficiency nonlinear‐optical mirror can be realized by our proposed spoof plasmonic metamaterial. According to the expression (1), we believe that the conversion efficiency of our work can be further improved by extending the nonlinear region of the spoof plasmonic metamaterial to increase the coherence length thanks to the ultralow loss of the CSPs, or improving the incident power *I_ω_* of the FF signal by optimizing the transition efficiency between the coplanar and the spoof plasmonic waveguides, as well as supplying the applied reverse voltage of the varactor to enhance the nonlinear susceptibility of the plasmonic waveguide.

In summary, we have experimentally verified the backward phase matching for SHG in a negative‐index nonlinear spoof plasmonic metamaterial with ultrathin geometry and ultralow loss at microwave frequencies. Numerical simulations and experimental results demonstrated that the backward phase matching for efficient SHG can be achieved by the negative‐index nonlinear spoof plasmonic metamaterial, whose design can be easily extended to terahertz regimes due to the geometrically induced spoof surface plasmons. Compared with the optical nonlinear processes, spoof plasmonic device has much lower dissipation loss as compared with its optical counterpart, enabling highly efficient SHG. Moreover, the backward phase‐matching condition can be realized at any frequency by simply changing the geometry of the plasmonic waveguide. We expect these results to be a necessary and vital step toward the realization of high efficiency nonlinear mirrors. Our study holds great promise for numerous practical applications in highly efficient nonlinear plasmonic components and devices in the context of complex integrated circuits and systems in the microwave and terahertz frequencies.

## 5 Experimental Section

*Numerical Simulation*: In numerical simulation of the dispersion relation for the nonlinear CSPs, the eigenmode solver in the commercial software of CST Microwave Studio could not analyze a structure containing lumped elements under periodic boundary conditions. To address it, a simplified finite‐volume lumped element method^45^ was employed to construct the varactor to get the simulated dispersion relation of the nonlinear CSPs. In numerical simulation of the surface electric‐field distributions, a diode lumped element with zero‐voltage capacitance (*C*_0_ = 2.35 pF) was added into each CSP unit cell, F4B (the relative permittivity ε_r_ = 2.55 and the loss tangent tanδ = 0.001) with thickness of 0.5 mm was used as the dielectric substrate material, which was a high‐quality printed circuit board commonly used in the microwave frequencies, and the transient solver in the CST Microwave Studio was employed. The quantitative propagation length of the symmetric and the antisymmetric nonlinear CSP modes were numerically calculated in the frequency domain using a commercial finite‐element solver (COMSOL Multiphysics^32^), and the experimentally measured optical properties of metal (copper^46^ in this case) was used to calculate the propagation length of the nonlinear CSPs.

*Spectrum Measurement*: The schematic diagram of a comprehensive setup to measure the SHG spectra has been sketched in Figure 3b and a photograph of the experimental prototype details is given in Figure S3 in the Supporting Information. A microwave signal generator (RoHDE&SCHWARZ SMB 100A) was employed to inject a series of FF signals into the nonlinear spoof plasmonic metamaterial and a spectrum analyzer (Agilent N9010A) was used to detect the SHG power spectra. In the measurement, the output port of the signal generator was connected to the input port (the positive pole of the varactor) of the fabricated sample through a sub‐miniature‐A (SMA) type connector to provide the incident FF signal. A 50 Ω broadband matched load was connected to the other port (the negative pole of the varactor) of the sample to reduce the significant back reflection inside the sample, preserving the directionality of the generated SH wave. Moreover, a low‐pass filter (LPF) with cut‐off frequency of 5.5 GHz was introduced between the sample and the signal generator to suppress the inherent higher harmonics from the signal generator. An electric monopole used as a detector was connected to the input port of the spectrum analyzer to probe the SHG spectra. The detecting probe was fixed 2 mm away from the sample and was always perpendicular to the sample surface. In order to show the advantage of the nonlinear conformal surface plasmonic waveguide over previous designs.^17^, ^18^ For example, its low‐loss property is quite robust to the bending angle θ, and then, we attached the fabricated sample to a θ =120° curved foam board. As shown in the measured results, although the nonlinear plasmonic waveguide was bent (θ = 120°), the generated backward SH waves had a similar propagation distance as that of θ = 180°. This property provided a feasible way for the future integration of the spoof plasmonic metamaterial designed with flexible electronics.

*Near‐Field Measurement*: The schematic diagram of the near‐field experiment system and a photograph were shown in Figure S4a,b in the Supporting Information. The near‐field measurement was conducted by using a near‐electric‐field scanning system, which was composed of a vector network analyzer (VNA, Agilent N5230C), source, detector, and motion controller. The fabricated sample was placed on the movable bottom metal plate, which could move in both the *x*‐ and *y*‐directions, controlled by the motion controller to achieve automated near‐electric‐field scanning with a step resolution of 0.5 mm. One port of the VNA was connected to the input port of the fabricated sample to excite the fundamental signal, and a same broadband matched load used in Figure S3 in the Supporting Information was connected to the other port of the sample. The same LPF used in Figure S3 in the Supporting Information was also employed between the sample and the VNA to filter the incident high‐frequency signals from the VNA. Due to the approximate quadratic relation of *I*_2ω_ ∝ (*I*_ω_)^2^ in Figure 5b, a power amplifier with gain of 23 dB was used between the LPF and the sample, intending to provide sufficient fundamental wave intensity to ensure the varactors working properly. An electric monopole connected to the other port of the VNA was fixed 3 mm above the sample to record the |Ez|‐component electric field on the observation *xy* plane with length *L_x_* = 40d = 140 mm along the *x*‐direction and width 40 mm along the *y*‐direction.

## Conflict of Interest

The authors declare no conflict of interest.

## Supporting information

Supplementary
