# Supplementary material for: Backward Phase Matching for Second Harmonic Generation in Negative‐Index Conformal Surface Plasmonic Metamaterials
Source: Adv Sci (Weinh). 2018 Aug 31;5(11):1800661. doi: 10.1002/advs.201800661 (PMC6247027; doi:10.1002/advs.201800661)
Supplement: Supplementary file 1 — Supplementary [file ADVS-5-1800661-s001.pdf]

Copyright WILEY-VCH Verlag GmbH & Co. KGaA, 69469 Weinheim, Germany, 2018.

ADVANCED SCIENCE Open Access

Supporting Information

for *Adv. Sci.,* DOI: 10.1002/advs.201800661

Backward Phase Matching for Second Harmonic Generation in Negative-Index Conformal Surface Plasmonic Metamaterials

Liangliang Liu, Lin Wu, Jingjing Zhang, Zhuo Li, Baile Zhang,* and Yu Luo**

Supporting Information

Backward Phase-Matching for Second Harmonic Generation in Negative-Index Conformal Surface Plasmonic Metamaterials

Liangliang Liu[1,2,3,#], Lin Wu[1,#], Jingjing Zhang[1,#], Zhuo Li[3,*], Baile Zhang[4,*], and Yu Luo[1,*]

[1]School of Electrical and Electronic Engineering, Nanyang Technological University, Nanyang Avenue, 639798, Singapore.

[2]the Research Center of Applied Electromagnetics, School of Electronic and Information Engineering, Nanjing University of Information Science and Technology, Nanjing, 210044,China.

[3]the Key Laboratory of Radar Imaging and Microwave Photonics, Ministry of Education, College of Electronic and Information Engineering, Nanjing University of Aeronautics and Astronautics, Nanjing 211106, China.

[4]Division of Physics and Applied Physics, School of Physical and Mathematical Sciences, Nanyang Technological University, Nanyang Avenue, 637371, Singapore.

[#]these authors contributed equally to this work.

*corresponding author: luoyu@ntu.edu.sg, blzhang@ntu.edu.sg, lizhuo@nuaa.edu.cn

1. Dispersion Relation of the Nonlinear CSP

In general, a transverse magnetic (TM) polarized incident EM wave with frequency ω_1 can excite a traditional CSP mode with frequency ω_1 at a time, as shown in **Figure S1(a)**, however, multifrequency EM waves mixing arising in a nonlinear process. To address it and to distinguish it from the traditional CSP, a nonlinear CSP is proposed and described in **Figure S1(b)**, in which a nonlinear active device (or component) is employed in each CSP unit cell structure. When a TM-polarized EM wave of frequency ω_1 incidents on the CSP structure, apart from the EM wave of frequency ω_1, multiple harmonic signals of frequency ω_2 ($\omega_2=n\omega_1$, n=2,3,......) with same or opposite propagation direction as the incident wave will be excited. In this case, the aforementioned nonlinear process can be proceeded.

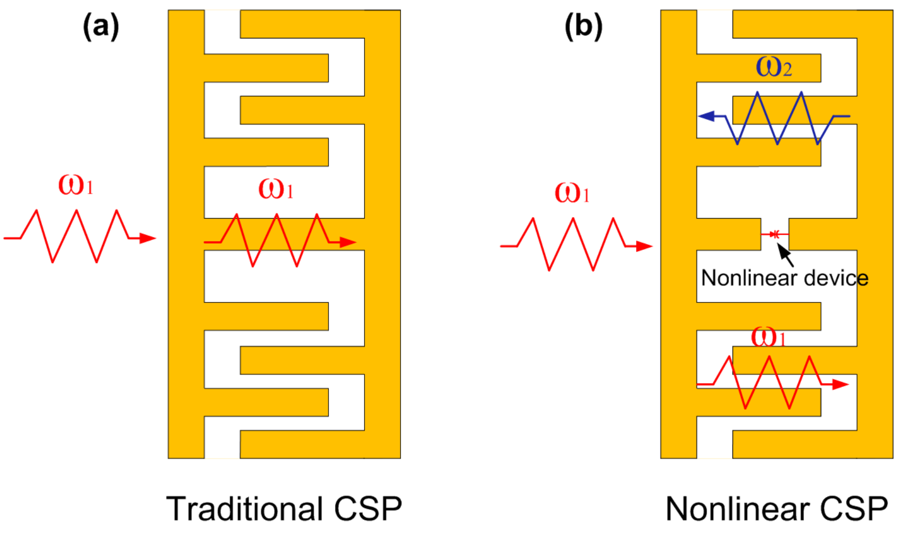

Supplementary Figure S1 | Schematic diagram of (a) traditional CSP and (b) nonlinear CSP unit cell structures.

As shown in **Figure S2(a)**, the dispersion curves corresponding to the traditional and nonlinear CSP are calculated through the eigenmode solver of the CST Microwave Studio. We observed that the dispersion curve of the nonlinear CSP is almost same as that of the traditional CSP, indicating the dispersion relation of the nonlinear CSP is rather insensitive to the structure with or without nonlinear device. When the capacitance value of the varactor is chosen as C=2.35 pF (the reverse voltage V_d=0V), 1.58 pF (V_d=1V), 0.683 pF (V_d=5V), and 0.497 pF (V_d=10V) respectively, we noticed that the dispersion relation of the nonlinear CSP is rather insensitive to the tunable capacitance values of nonlinear device, as shown in **Figure S2(b)**.

One of the most important advantages of the proposed nonlinear spoof plasmonic metamaterial is the ultra-low transmission loss. **Figure S2(d)** presents the numerically calculated quantitative propagation length of the symmetric and the antisymmetric CSP modes. We observe that both them have a long propagation length, i.e. 88λ for f_{FF}=3.62 GHz in the symmetric mode and -52λ for f_{SH}=7.24 GHz in the antisymmetric mode. It is noteworthy that the emergence of the negative propagation length is due to the negative group velocity of the antisymmetric mode and the introduced calculation formula of the propagation length[26,28] $L=Q\times v_g/\mathrm{Re}(\omega)$ in the case. Here, $v_g=d\omega/dk$ is obtained by the derivative of the dispersion curve of the CSP mode and is shown in **Figure S2(c)**, the quality factor Q in the periodical structure is calculated by $Q=\mathrm{Re}(\omega)/[-2\mathrm{Im}(\omega)]$, where Re(ω) and Im(ω) denote the real and imaginary parts of the complex eigen-frequency respectively.

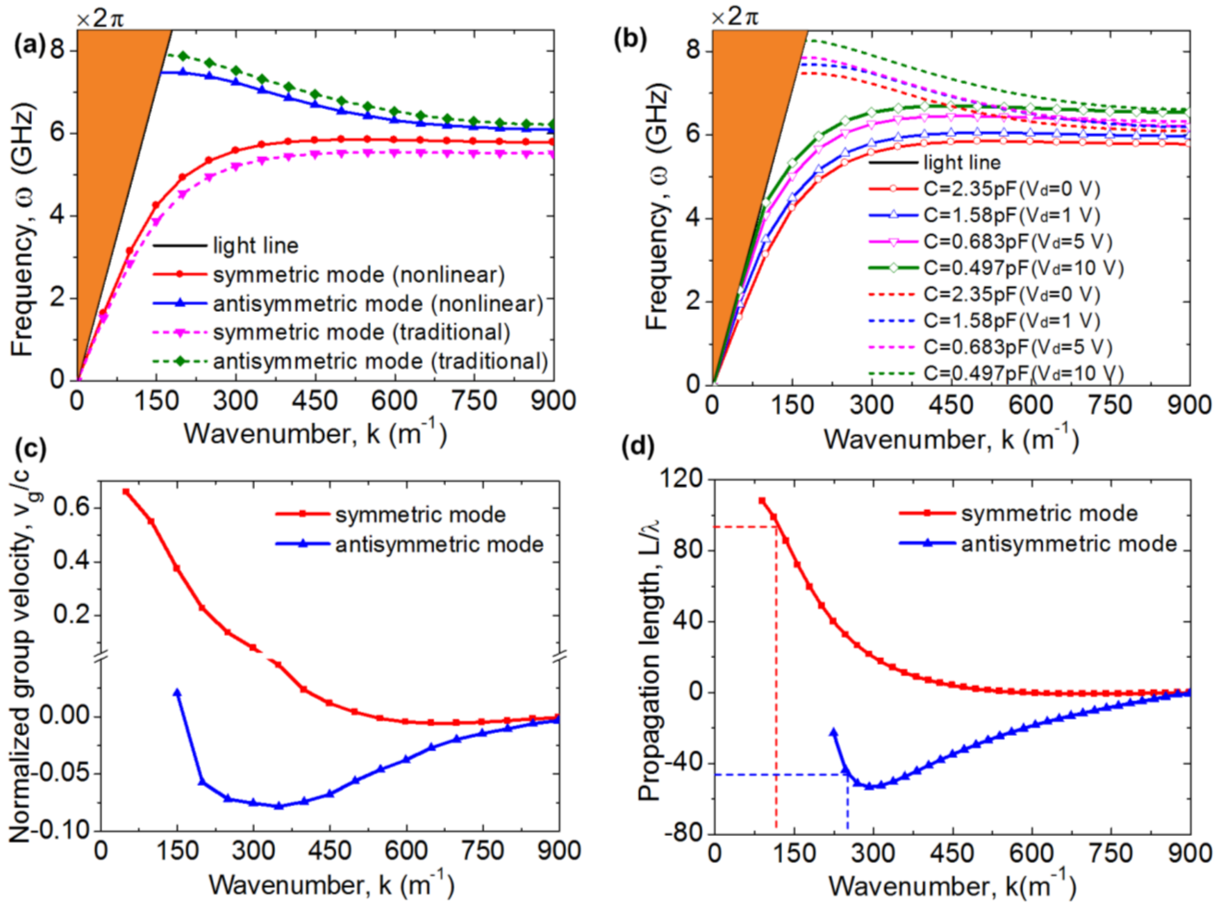

Supplementary Figure S2 | (a) Comparison of the dispersion relation for the traditional and nonlinear CSPs. (b) The evolution of the dispersion relation for the nonlinear CSP when the varactor varies with different capacitance values. (c) Normalized group velocity (v_g/c, c is the light velocity in free space) and (d) propagation length of the symmetric and the antisymmetric modes.

2. The Spectrum and Near-Field Measurement Systems.

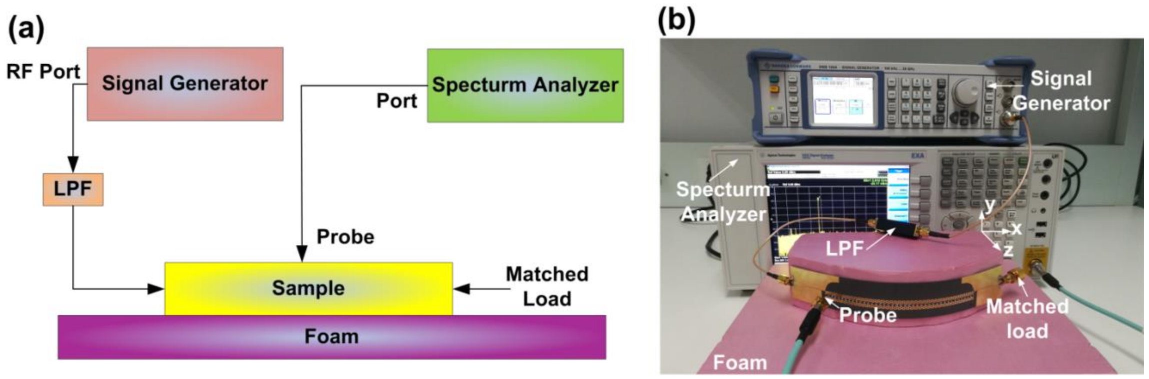

Supplementary Figure S3 | (a) The schematic diagram for spectrum measurement and (b) the corresponding photograph of the experimental prototype respectively.

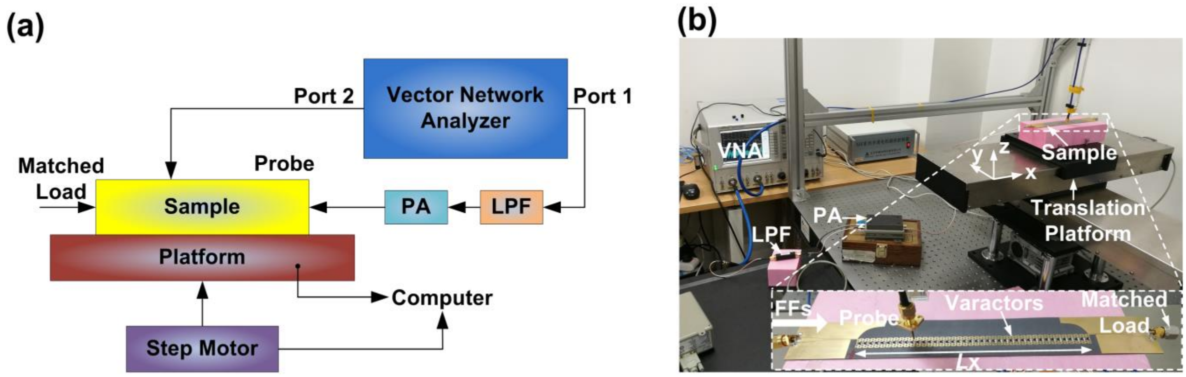

Supplementary Figure S4 | (a) The schematic diagram for near-field measurement and (b) the corresponding photograph of the experimental apparatus respectively. Inset: the enlarge view of the fabricated sample pasted on the surface of a foam broad.
